# *NOTCH1* Gene as a Novel Cause of Thoracic Aortic Aneurysm in Patients with Tricuspid Aortic Valve: Two Cases Reported

**DOI:** 10.3390/ijms24108644

**Published:** 2023-05-12

**Authors:** Laura Torres-Juan, Yolanda Rico, Elena Fortuny, Jaume Pons, Rafael Ramos, Fernando Santos-Simarro, Víctor Asensio, Iciar Martinez, Damian Heine-Suñer

**Affiliations:** 1 Molecular Diagnostics and Clinical Genetics Department (UDMGC), Hospital Universitari Son Espases, 07010 Palma de Mallorca, Spain; laura.torresjuan@ssib.es (L.T.-J.); fernando.santos@ssib.es (F.S.-S.); victor.asensio@ssib.es (V.A.); iciar.martinez@ssib.es (I.M.); 2 Health Research Institute of the Balearic Islands (IdISBa), Hospital Universitari Son Espases, 07010 Palma de Mallorca, Spain; elena.fortuny@ssib.es (E.F.); jaumea.pons@ssib.es (J.P.); rafaelf.ramos@ssib.es (R.R.); 3 Cardiology Department, Hospital Universitari Son Espases, 07010 Palma de Mallorca, Spain; yolanda.rico@ssib.es; 4 Pathology Department, Hospital Universitari Son Espases, 07120 Palma de Mallorca, Spain

**Keywords:** *NOTCH1*, *MIB1*, thoracic aortic aneurysm, deletion, tricuspid aortic valve, Notch pathway

## Abstract

Thoracic aortic aneurysms (TAA) consist of abnormal dilation or the widening of a portion of the ascending aorta, due to weakness or destructuring of the walls of the vessel and are potentially lethal. The congenital bicuspid aortic valve (BAV) is considered a risk factor for the development of TAA because asymmetric blood flow through the bicuspid aortic valve detrimentally influences the wall of the ascending aorta. *NOTCH1* mutations have been associated with non-syndromic TAAs as a consequence of BAV, but little is known regarding its haploinsufficiency and its relationship with connective tissue abnormalities. We report two cases in which there is clear evidence that alterations in the *NOTCH1* gene are the cause of TAA in the absence of BAV. On the one hand, we describe a 117 Kb deletion that includes a large part of the *NOTCH1* gene and no other coding genes, suggesting that haploinsufficiency can be considered a pathogenic mechanism for this gene associated with TAA. In addition, we describe two brothers who carry two variants, one in the *NOTCH1* gene and another in the *MIB1* gene, corroborating the involvement of different genes of the Notch pathway in aortic pathology.

## 1. Introduction

Thoracic aortic aneurysms (TAA) consist of abnormal dilation or the widening of a portion of the ascending aorta, due to weakness or destructuring of the walls of the vessel. TAA are potentially lethal and represent between 1–2% of the causes of death in industrialized countries [1]. To date, TAA pathogenesis is still poorly understood. The most widely accepted causes involve genes which encode proteins that are part of the extracellular matrix, the transforming growth factor beta signaling pathway or participate in smooth muscle cell contraction [2].

On the other hand, the congenital bicuspid aortic valve (BAV) is considered a risk factor for the development of TAA both for mechanical reasons and for genetic reasons. First, because asymmetric blood flow through the bicuspid aortic valve detrimentally influences the wall of the ascending aorta [3], and second, some cases have also been reported in which a genetic defect is the cause of BAV formation and aortic dilation. Accordingly, 32% of first-degree relatives of BAV patients also develop aortic root dilatation, suggesting that there is a common genetic predisposition for BAV and TAA [4]. However, some authors suggest that TAA in BAV and tricuspid aortic valve (TAV) patients arise from different molecular, cellular and genetic mechanisms [5,6].

Aneurysms of the ascending aorta can be divided into familial and non-familial and further subdivided into syndromic or non-syndromic. Familial non-syndromic TAAs associated with BAV can be caused by *NOTCH1* among other genes [2]. The *NOTCH1* gene is located on chromosome 9q34 and provides instructions for making the Notch1 protein, a transmembrane receptor that, together with three other receptors of the same family (Notch2, Notch3 and Notch4), are part of a highly conserved signaling pathway. The Notch signaling pathway plays a critical role during mammalian cardiac development [7] and, mutations in genes of this pathway are associated with several congenital disorders involving malformed valves, the aortic arch, or a defective chamber septation [8]. *NOTCH1* pathogenic variants have been associated not only with non-syndromic TAAs but also with conotruncal congenital heart defects and complex syndromes such as Adams–Oliver [9,10,11]. Based on a mouse model, haploinsufficiency may be a pathogenic mechanism for TAAs associated with the *NOTCH1* gene, as heterozygous knockout mice developed aortic root dilation [12]. However, de novo deletions containing the *NOTCH1* gene have only been described in patients with congenital heart defects such as tetralogy of Fallot (TOF), hypoplastic left heart syndrome (HLHS), and ventricular septal defect, but not in non-syndromic TAAs and tricuspid aortic valve (TAV). Furthermore, in most described cases, contiguous genes were also deleted which may have contributed to the cardiac phenotype [13].

Currently, many efforts are being made to understand the role of the *NOTCH1* gene in congenital heart defects. A recent study carried out with experiments in human cells determines how mutations in the *NOTCH1* gene impact cardiac cell differentiation and proliferation. Specifically, single-cell transcriptomic analysis reveals the abnormal cell lineage specification and imbalanced differentiation of the first heart field, second heart field and epicardial progenitors in *NOTCH1*-deficient induced pluripotent stem cells [14].

We present two cases with TAA and a tricuspid aortic valve that present alterations involving the *NOTCH1* gene and, therefore, propose that the alteration of this gene is a novel cause of TAA alone and not as a result of the BAV. In addition, we document in one of the two cases, a deletion that affects exclusively the *NOTCH1* gene associated with TAA suggesting that haploinsufficiency can be considered a pathogenic mechanism for this gene.

## 2. Results

### 2.1. Case 1

**Figure 1:**
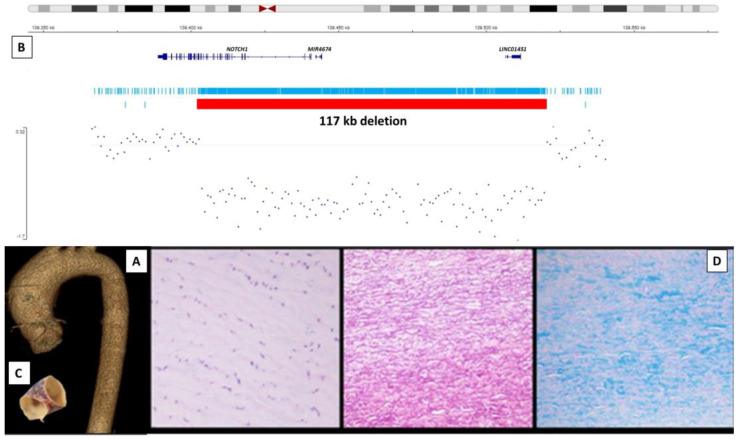
Case 1: (**A**) 3D reconstruction of the thoracic aorta showing ascending aortic aneurysm. (**B**) Deletion of 117 kb at 9q34.3 detected by whole genome sequence which includes large part of the *NOTCH1* gene (from the start of the gene to exon 18) as well as non-coding RNA genes (MIR4673, MIR4674, NALT1 y LINCO1451). (**C**) Aortic artery segment of 2.6 cm with an average increased diameter of 3–3.1 cm and thinned wall. (**D**) Histological findings in the middle layer of the aortic wall, from left to right: hematoxylin-eosin showing middle layer degeneration with loss of cellularity (×200), fragmented elastic fibers (orcein ×100) and cystic necrosis (alcian-blue ×100).

The first case is a 43-year-old woman initially sent to cardiology because of a suspicion of Marfan syndrome (MS) due to pectus carinatum, increased arm span/height and scoliosis. The patient did not reach diagnostic criteria for MS with a systemic score < 7 points (revised Ghent nosology) but the tricuspid aortic valve with mild double lesion and no aortic dilatation was observed in the transthoracic echocardiogram (TTE), so a regular follow-up was recommended. The patient missed follow-ups for eight years, and an updated TTE was performed because of the new onset of fatigue and dyspnea showed ascending aortic dilation of 43 mm, mitral valvular prolapse with mild mitral regurgitation and double aortic lesion with light stenosis and severe regurgitation, resulting in moderate left ventricular enlargement (Figure 1A).

The patient underwent successful cardiac surgery with Bentall–Bono technique and a highly unstructured tricuspid aortic valve and severe fragility of the aortic tissue were reported by surgeons. Histological study showed thinning with intense degeneration of the middle layer characterized by alteration of smooth muscle fibers, fragmentation, loss and disorganization of elastic fibers and accumulation of basophilic mucoid material with areas of fibrosis and no associated inflammatory signs. These are the typical alterations of what is called cystic medial necrosis, which characterizes non-inflammatory degenerative aneurysms of the aorta (Figure 1C,D). No clinical complications have appeared after two years of follow-up, even though we are closely monitoring the pulmonary artery dilatation of 40 mm detected in the last thoracic scan.

As part of the study of complex aortic pathology, a genetic study was indicated. WGS detects single nucleotide variants, deletions and duplications in all the genome sequences and allows us to characterize non-coding and regulatory as well as coding variants. It confirmed the *NOTCH1* deletion but did not find any other potentially pathogenic variants related to the phenotype. Our results show that the deletion measures 117 kb at 9q34.3 (chr9:136.508.773–136.625.728 (GCRh38)) and includes a large part of the *NOTCH1* gene (from the 5′-UTR to exon 18) and no other coding genes (Figure 1B). Unfortunately, it was not possible to study her parents since they were deceased, although it was documented that her father had heart surgery and was a prosthetic aortic valve carrier. Her sister, who was examined for aortic pathology and found to be unaffected, does not carry the deletion.

### 2.2. Case 2

**Figure 2:**
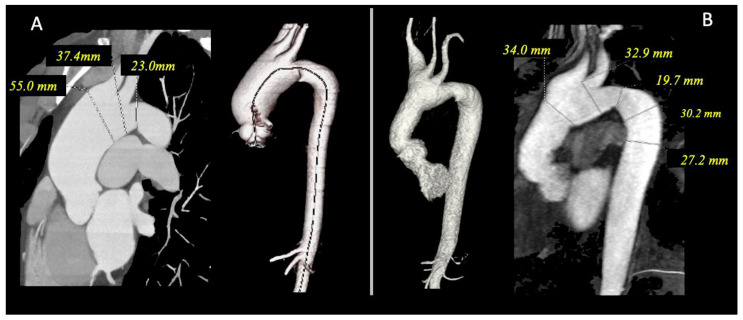
Case 2: (**A**) Chest CT angiography showing aneurysm of the ascending aorta (55 mm) with tricuspid aortic valve, bovine aortic arch and dilatation of right brachiocephalic trunk. (**B**) Postoperative angio-MRI with normal size of ascending aorta.

The second case is a 48-year-old man with a normal complexion diagnosed with a non-syndromic aneurysm of the ascending aorta (maximum 55 mm) with a tricuspid aortic valve (Figure 2A). He underwent elective aortic surgery with the interposition of a prosthetic tube graft. The anatomopathology of the aortic wall sample showed cystic degeneration. The patient also presented a bovine aortic arch and dilatation of the right brachiocephalic trunk and main pulmonary arteries (38 mm), with the stability of these findings in the imaging test that has been performed over nine years (Figure 2B).

A genetic study was indicated and an NGS panel of 30 genes detected the presence of two variants, one in the *NOTCH1* gene and another in the *MIB1* gene.

The *NOTCH1* (NM_017617.5) variant: c.844C > T p. Arg282Cys is located in exon 5 and has not been previously described. It has been detected in 1 of 247,992 chromosomes in the general population (according to the GnomAD database). Computational predictors assign a probable pathogenic effect with a score REVEL = 0.8579 [15], supported because the position is strongly conserved (phyloP100way = 7.82). Despite this, following the criteria for the classification of variants recommended by the ACMG, it is classified as a variant of uncertain significance (PM2, PP3) [16].

In addition, this patient is also a carrier of a *MIB1* (NM_020774.4) variant: c.971_972del p.Gln324ArgfsTer13 located in exon 7. This is a null variant (frameshift) so it should supposedly give rise to a truncated protein. This variant has not been previously described and is not found in the general population databases (according to the GnomAD database). Following the criteria for the classification of variants recommended by the ACMG, it is classified as a likely pathogenic (PVS1, PM2) [16].

These two variants were then studied in the patient’s two healthy children who were found to be non-carriers. Subsequently, his older brother was also studied, finding in the imaging test a slight dilation of the aorta with a low rate of dilation (ascending aorta in 2017 of 36 mm and in 2021 of 40 mm). The genetic study of the two variants found in the brother revealed that he was a carrier of both, so a close follow-up is being performed.

## 3. Discussion

Several studies conclude that the molecular mechanisms that promote the development of aneurysms of the ascending aorta in the presence of a BAV are different from that in patients with a TAV [5,17,18]. In support of this, genes that have been associated with BAV to date, are rarely altered when BAV is accompanied by TAA. Furthermore, the cases presented here support the hypothesis that alterations in the *NOTCH1* gene can be associated with non-syndromic TAA in the absence of BAV.

It is known that the Notch signaling pathway plays an important role in the development of the cardiovascular system and recent studies determine that Notch signaling plays an important role in the pathogenesis of the TAA [18]. The *NOTCH1* gene encodes a transmembrane notch receptor. It is a protein that serves as a receptor for extracellular signals and participates in several signaling pathways during development. Several publications have associated pathogenic variants in *NOTCH1* with aortic pathology. Garg V et al. found that *NOTCH1* is expressed in the developing cardiac outflow tract, and pathogenic variants in *NOTCH1* were related to aortic valve disease (BAV) associated with severe valve calcification or dilation and aneurysm of the aorta [19,20,21]. Subsequently, other groups have reported cases of patients with variants in *NOTCH1* who presented aortic coarctation or aortic stenosis and TAV [22,23,24]. Finally, in another study, thoracic aortic aneurysms (TAA) have been described in six individuals with *NOTCH1* variants, and these were associated with BAV in four and with severe aortic valve stenosis (AVS) in the other two cases [11]. To our knowledge, inactivating *NOTCH1* mutations leading to *NOTCH1* haploinsufficiency have almost exclusively been reported associated with aortic valve disease [11,25]. However, in studies with mice, there are indications that haploinsufficiency of the *NOTCH1* gene can contribute to the aortic pathology and the development of TAA. Koenig et al. generated *NOTCH1*-haploinsufficient mice without BAV characteristics that exhibited aortic root dilation indicating that loss of Notch1 is sufficient to cause TAA [12].

Vascular smooth muscle cells of the ascending aorta are responsible for the secretion of factors associated with aneurysm formation [26]. Reviewing the embryonic origin of the cells that form the aortic valve and the ascending aorta, it is known that smooth muscle cells of the aortic valve arise from the second cardiac field, while those of the ascending aorta form an inner layer of smooth muscle cells come from the neural crest and an outer layer of cells come from the second cardiac field [18]. Therefore, cells that come from the second cardiac field will be part of both the aortic valve and the ascending aorta. In fact, High et al. showed that either absence of the Notch ligand Jagged1 or inhibition of Notch signaling in second heart field tissues results in the murine aortic arch artery and cardiac anomalies [27]. In addition, a very recent study using CRISPR/Cas9 genome editing technology to delete *NOTCH1* in human-induced pluripotent stem cells, reveals that *NOTCH1* modulates cell fate determination of early cardiac mesoderm towards the first heart field, second heart field and epicardial progenitors [14]. However, more experiments are needed to demonstrate that the dysregulation of *NOTCH1* in cells of the second heart field during embryonic development leads to malformations in the aortic valve or dilation of the ascending aorta.

On the other hand, Notch activation requires the endocytosis of Notch ligands in the signal-sending cells. MIB1 is an E3 ubiquitin ligase that regulates endocytosis of Notch ligands [28]. *MIB1* mutations reduce Notch signaling activation and contribute to congenital heart disease [29]. Interestingly, Luxan G et al. show that germline mutations in human *MIB1* cause left ventricular noncompaction cardiomyopathy in autosomal-dominant pedigrees, with affected individuals showing reduced *NOTCH1* activity and reduced expression of target genes [30].

In this paper, we present two cases in which a potentially reduced activity of the *NOTCH1* gene, due to haplinsufficiency and a dysregulation of the endocytosis of its ligands, can favor the development of TAA in patients in the presence of a normal aortic valve (TAV).

In case 1, different to other previous studies, the only coding gene that is impacted by the haploinsufficiency is *NOTCH1*, ruling out the involvement of other coding genes in the phenotype. Concurrently, as in patients described previously with larger deletions, our patient presents involvement of the aorta. In the same manner, a patient with an 85Kb deletion affecting solely the promoter region and exon 1 of *NOTCH1* has an Adams–Oliver Syndrome phenotype similar to other patients with nonsense mutations in *NOTCH1* (Table 1). Here, we associate a deletion affecting exclusively the *NOTCH 1* gene with non-syndromic aortic pathology, supporting that haploinsufficiency of *NOTCH1* is sufficient to cause aortic dilation.

In case 2, we introduce two brothers with non-syndromic TAA and tricuspid aortic valves showing different degrees of aortic dilation, and both presented with two variants in *NOTCH1* and *MIB1*. This case, in addition to corroborating the involvement of different genes of the Notch pathway in aortic pathology, also indicates that these alterations have variable expressivity, as both brothers (carrying the same genetic variants) have different degrees of aortic and great vessel dilation. It would be interesting in the future to carry out functional studies to understand the degree of participation of each of these genes in the pathology.

Up to now, alterations in the *NOTCH1* gene have been associated with aortic valve pathology, conotruncal heart defects and Adams–Oliver syndrome (OMIM * 190198) but are not considered to cause TAA with TAV. Although two cases are not enough to draw a definitive and reliable conclusion, this study is an invitation to explore the *NOTCH1* gene and the genes involved in its regulation as a new cause of thoracic aortic aneurysm in the absence of BAV.

## 4. Materials and Methods

Informed consent was obtained from all patients under the institutional review board policies of the hospital. Diagnostic criteria for TAA were met because of phenotypic expression and familial background. Accordingly, we performed an exhaustive family history screening for aortic pathology of all living first-degree relatives and a genetic study of the proband using next generation sequencing technique.

For the genetic study, genomic DNA was extracted through peripheral blood leukocytes according to standard protocols.

Case 1 was studied in our laboratory by Next Generation Sequencing (NGS) of the clinical exome (Trusight One Expanded© panel from Illumina) which includes ~16.5 Mb of genomic content and 6700 genes that have been associated with human pathology. The library generated with this panel was sequenced on an Illumina NextSeq platform to ~120× depth of reads.

Clinical exome analysis using the DRAGEN v4.0.3 alignment pipeline and the Geneyx software v5.5a suggested a deletion in the NOTCH1 gene. To confirm this, a whole genome sequence (WGS) was performed using the Illumina TruSeq PCR-free kit, 2 × 150 on NovaSeq S4 v1.5. Variant analysis was performed in our laboratory using the Trusight Software Suite (TSS) v.2.0.2 from Illumina and Geneyx.

Case 2 was studied by Health in Code (A Coruña, Spain) by NGS using a library that includes 30 genes related to hereditary cardiovascular disease. The study includes the analysis of all coding exons and flanking intronic regions of these genes.

## Figures and Tables

**Table 1:** Phenotype–genotype comparison between patients previously described carrying a less than 1Mb deletion that includes *NOTCH 1* gene and our patients.

Patient	Cardiac Phenotype	Deletion Size	Genes Included in the Deletion
A2 *	Bicuspid aortic valve	0.22 Mb	Three OMIM Morbid map genes (NOTCH1, INPP5E and PMPCA) and two RefSeq genes (SEC16A and C9orf163)
A3 *	Bicuspid aortic valve and coarctation of the aorta, with normal left ventricular size, morphology and function.	0.22 Mb	Three OMIM Morbid map genes (NOTCH1, INPP5E and PMPCA) and two RefSeq genes (SEC16A and C9orf163)
B1 *	Hypoplastic left heart syndrome, dysplastic mitral valve, double outlet RV and tubular hypoplasia of the left aortic arch.	614.3 kb	NOTCH1 gene and three other OMIM morbid map genes (AGPAT2, ABCA2 and MAN1B1). The deletion also contained 22 RefSeq genes (SEC16A, EGFL7, MIR126, LCN10, LCN6, LCN8, C9orf86, PHPT1, EDF1, TRAF2, FBXW5, C8G, LCN12, PTGDS, CLIC3, ABCA2, FUT7, NPDC1, ENTPD2 and C9orf140).
1-II-3 **	Adams–Oliver Syndrome with narrow pulmonary arteries	85 kb	5′ region of NOTCH1 (Promotor and exon 1)
Our patient, case 1	Tricuspid aortic valve with light stenosis and severe regurgitation, moderate left ventricular dilatation, mitral valvular prolapse, ascending aortic dilation of 43 mm and pulmonary artery dilatation	117 kb	Large part of the NOTCH1 gene (up to exon 18) as well as three non-coding RNA genes (MIR4673, MIR4674, NALT1 y LINCO1451).

* Patients described by Roifman M et al. [13], ** Patients described by Stittrich AB et al. [9].

## Data Availability

The data that support the findings of this study are available from the corresponding author upon reasonable request.
